# Supplementary material for: Perioperative Blood Transfusion Is Dose-Dependently Associated with Cancer Recurrence and Mortality after Head and Neck Cancer Surgery
Source: Cancers (Basel). 2022 Dec 23;15(1):99. doi: 10.3390/cancers15010099 (PMC9817502; doi:10.3390/cancers15010099)
Supplement: Supplementary file 1 [file cancers-15-00099-s001.zip › Supplementary Table S1.pdf]

Supplementary Table S1 Results of logistic regression analysis for inverse probability treatment weighting

	OR	95% CI	p
Age	1.02	1.00 ~ 1.05	0.090
Sex (female vs. male)	0.97	0.34 ~ 2.75	0.953
BMI	0.97	0.91 ~ 1.04	0.390
ASA > 3	0.88	0.49 ~ 1.61	0.687
Smoking	0.77	0.31 ~ 1.92	0.569
Betel nut chewing	2.26	1.09 ~ 4.69	0.029
Alcohol	0.83	0.44 ~ 1.58	0.569
Preoperative hemoglobin	0.67	0.56 ~ 0.80	< 0.001
Anesthesia time*	2.94	1.99 ~ 4.34	< 0.001
Blood loss during surgery*	1.97	1.62 ~ 2.40	< 0.001
Positive surgical margin	0.59	0.32 ~ 1.08	0.088
Primary tumor (T3,4 vs. T1,2)	2.08	1.11 ~ 3.88	0.021
Lymph node involvement	1.76	0.95 ~ 3.25	0.072
Histologic differentiation (Moderate to severe vs. well)	0.92	0.54 ~ 1.56	0.754
Adjunct radiotherapy	0.99	0.34 ~ 2.84	0.983

Adjunct chemotherapy	0.83	0.30 ~ 2.33	0.725

OR: odds ratio; BMI: body mass index; ASA: ASA physical status. *On base-2 logarithmic scale
