# Supplementary material for: Perioperative Blood Transfusion Is Dose-Dependently Associated with Cancer Recurrence and Mortality after Head and Neck Cancer Surgery
Source: Cancers (Basel). 2022 Dec 23;15(1):99. doi: 10.3390/cancers15010099 (PMC9817502; doi:10.3390/cancers15010099)
Supplement: Supplementary file 1 [file cancers-15-00099-s001.zip › Supplementary Table S2.pdf]

Supplementary Table S2 Frequency table of perioperative packed red blood cell transfusion in surgery for head and neck cancer

Unit	Frequency	%	Cumulative %
0	491	71.9	71.9
1	3	0.4	72.3
2	49	7.2	79.5
3	3	0.4	79.9
4	53	7.8	87.7
6	42	6.1	93.9
7	4	0.6	94.4
8	27	4.0	98.4
9	1	0.1	98.5
10	6	0.9	99.4
11	1	0.1	99.6
12	2	0.3	99.9
22	1	0.1	100.0
