# Supplementary material for: Perioperative Blood Transfusion Is Dose-Dependently Associated with Cancer Recurrence and Mortality after Head and Neck Cancer Surgery
Source: Cancers (Basel). 2022 Dec 23;15(1):99. doi: 10.3390/cancers15010099 (PMC9817502; doi:10.3390/cancers15010099)
Supplement: Supplementary file 1 [file cancers-15-00099-s001.zip › Supplementary Table S3.pdf]

Supplementary Table S3 Distribution of occurred second primary cancers after surgery for head and neck cancer

Site	Frequency	%	Cumulative %
Head and neck	59	59.0	59.0
Esophagus	20	20.0	79.0
Others	21	21.0	100.0
Total	100		
